# A Randomized Controlled Trial Protocol to Evaluate the Effectiveness of an Integrated Care Management Approach to Improve Adherence Among HIV-Infected Patients in Routine Clinical Care: Rationale and Design

**DOI:** 10.2196/resprot.5492

**Published:** 2016-10-05

**Authors:** Heidi M Crane, Rob J Fredericksen, Anna Church, Anna Harrington, Paul Ciechanowski, Jennifer Magnani, Kari Nasby, Tyler Brown, Shireesha Dhanireddy, Robert D Harrington, William B Lober, Jane Simoni, Stevan A Safren, Todd C Edwards, Donald L Patrick, Michael S Saag, Paul K Crane, Mari M Kitahata

**Affiliations:** ^1^ Department of Medicine University of Washington Seattle, WA United States; ^2^ Department of Psychiatry University of Washington Seattle, WA United States; ^3^ Department of Psychology Harvard University Boston, MA United States; ^4^ Department of Health Services University of Washington Seattle, WA United States; ^5^ Department of Medicine University of Alabama, Birmingham Birmingham, AL United States

**Keywords:** adherence, randomized controlled trial, depression, substance use, alcohol use, intervention, HIV, care management

## Abstract

**Background:**

Adherence to antiretroviral medications is a key determinant of clinical outcomes. Many adherence intervention trials investigated the effects of time-intensive or costly interventions that are not feasible in most clinical care settings.

**Objective:**

We set out to evaluate a collaborative care approach as a feasible intervention applicable to patients in clinical care including those with mental illness and/or substance use issues.

**Methods:**

We developed a randomized controlled trial (RCT) investigating an integrated, clinic-based care management approach to improve clinical outcomes that could be integrated into the clinical care setting. This is based on the routine integration and systematic follow-up of a clinical assessment of patient-reported outcomes targeting adherence, depression, and substance use, and adapts previously developed and tested care management approaches. The primary health coach or care management role is provided by clinic case managers allowing the intervention to be generalized to other human immunodeficiency virus (HIV) clinics that have case managers. We used a stepped-care approach to target interventions to those at greatest need who are most likely to benefit rather than to everyone to maintain feasibility in a busy clinical care setting.

**Results:**

The National Institutes of Health funded this study and had no role in study design, data collection, or decisions regarding whether or not to submit manuscripts for publication. This trial is currently underway, enrollment was completed in 2015, and follow-up time still accruing. First results are expected to be ready for publication in early 2017.

**Discussion:**

This paper describes the protocol for an ongoing clinical trial including the design and the rationale for key methodological decisions. There is a need to identify best practices for implementing evidence-based collaborative care models that are effective and feasible in clinical care. Adherence efficacy trials have not led to sufficient improvements, and there remains little guidance regarding how adherence interventions should be implemented into clinical care. By focusing on improving adherence within care settings using existing staff, routine assessment of key domains, such as depression, adherence, and substance use, and feasible interventions, we propose to evaluate this innovative way to improve clinical outcomes.

**Trial Registration:**

Clinicaltrials.gov NCT01505660; http://clinicaltrials.gov/ct2/show/NCT01505660 (Archived by WebCite at http://www.webcitation/ 6ktOq6Xj7)

## Introduction

Adherence to antiretroviral medications (ARVs) is a key determinant of outcomes including viral suppression and prevention of disease progression and death [1-9]. Unfortunately, poor adherence among persons living with human immunodeficiency virus (HIV/PLWH) is common with mean levels of adherence in clinical cohorts often 60% to 80% or less [3,10-13]. Substance use and mental illnesses such as depression are key predictors of poor adherence [3,10,14-36], common among PLWH [20,36-41] and may be crucial to identify and treat among those with poor adherence [42].

The importance of translating research on improving adherence into clinical practice has been noted [43]. Previous studies investigating a variety of adherence interventions have reported benefits, although often small [44-46]. Many focused on testing a single device, such as a pager or other electronic reminder system, or investigated the effects of time-intensive costly interventions that are not feasible in most clinical settings, and therefore failed to inform adherence in clinical care [44].

We sought to evaluate a collaborative care approach as a feasible intervention applicable to PLWH in care, including those with mental illness and substance use. Collaborative care approaches are multimodal interventions that typically involve a care manager who helps develop a shared definition of a problem, sets goals, develops specific action plans, offers problem solving and support, and facilitates appointments [47]. These interventions have shown value for people with depression and anxiety, heart failure, and chronically ill seniors [47-62]. While there is little experience with care management approaches among PLWH particularly as related to adherence, treatment of depression is an exception [63]. Many studies of care management models have been conducted as off-site studies separate from clinic settings, have incorporated extensive additional clinical research staff, or have included time-intensive interventions such as community outreach with home visits that may not be feasible to implement on a broad scale in clinical care [49-51,63].

We developed a randomized controlled trial (RCT) investigating an integrated clinic-based care management approach to improve outcomes. This approach is based on routine integration and systematic follow-up of a clinical assessment of patient-reported outcomes (PROs) targeting domains, including adherence, depression, and substance use, and adapts the previously tested care management approach used in the Program to Encourage Active, Rewarding Lives (PEARLS) study [50]. The health coach or care management role is provided by clinic case managers allowing the intervention to be generalizable to other HIV clinics with case managers. We hypothesize that our care management intervention will improve adherence compared with usual care. This paper describes the trial design and rationale for key methodological decisions.

## Methods

### Description of Trial and Intervention

#### Overview

We integrated a RCT into clinical care of PLWH. PLWH who reported inadequate adherence on the clinical assessment of PROs that is already being completed as part of routine clinical care visits were eligible for the RCT. Case managers in the clinic serve as the care managers for the intervention arm of the study. They are already part of the clinic and available to help PLWH when requested. However, in addition to these services, those in the intervention arm received a more structured and scheduled care management intervention, that included scheduled assessments and follow-up, a stepped-care approach, and, if needed, more intensive intervention such as problem solving therapy. A key difference between the intervention and usual care arms is that the case managers receive automatic email reminders of the need to follow-up with the patient at set intervals and conduct specific assessments facilitating a more systematic approach rather than ad hoc support.

#### Design

This prospective RCT is integrated into clinical care of PLWH at the University of Washington (UW) Harborview Madison HIV clinic (hereinafter “the UW HIV clinic”). Eligible patients are randomized to either a usual care arm or an intervention/enhanced support arm with a 1:1 ratio without blocking or stratification using a computer-based random number generator. Results of the randomization are applied to the database and tracking platform by a research coordinator who oversees the computer-based number generator and is not involved in enrollment and consent and has not met the participants. Patients are followed for 12 months as part of the trial.

#### Setting

The UW HIV clinic is the largest single provider of medical care to PLWH in the northwestern United States and provides care to approximately 2800 PLWH. The clinic provides primary care, on-site specialty care, financial and case management, and pharmacy services.

#### Clinical Assessment

We developed and implemented a clinical assessment platform for routine collection of PROs in clinical care, including instruments that measure medication adherence, drug and alcohol use, sexual risk behavior, and depression and anxiety symptoms [64-68]. Examples of instruments collected as part of the clinical assessment include the 9-item Patient’s Health Questionnaire (PHQ-9) measure of depression symptoms [69,70], medication adherence (several items with varying recall periods) [71,72], and substance use (Alcohol Use Disorders Identification Test Consumption Items and Alcohol, Smoking, and Substance Involvement Screening Test) [73-76]. We obtained input from outside clinical technology experts and conducted time-and-motion studies and qualitative semistructured interviews from key informants including patients, providers, and staff members to ensure implementation was not disruptive and inform the content of the assessment [65]. A primary goal was to ensure that the clinical assessment was completed on the day of and prior to provider visits to ensure that assessment feedback was available to providers at the time of the visit [77]. Patients at the UW HIV clinic complete the assessment every 4 to 6 months and assessment feedback is given to providers and case managers as a now established part of clinical care. This occurs for all patients regardless of whether or not they are in this trial.

#### Participants

Participants are 18 years of age or older, English-speaking PLWH with inadequate ARV adherence as measured by self-report using a single item on the clinical assessment given to all patient at Madison clinic asking about the number of missed doses during the prior 14 days. Eligible patients have access to either a home or cellphone and are in care at the UW HIV clinic for at least 6 months. Patients are ineligible if they are severely cognitively impaired or actively psychotic as these patients are not asked to complete the clinical assessment. To enhance generalizability and relevance to a broad spectrum of patients in routine care, patients are not excluded based on substance use or depression.

#### Intervention

The intervention uses a care management approach that is incorporated into clinical care settings and has previously been shown to be useful in the care of patients with diabetes and epilepsy and more effective than providing PRO feedback alone [47,50,51,54]. Case managers are tasked with providing patient education and support, working with patients to develop a shared focus on specific problems with targeted goals and specific action plans. Our choice to incorporate case managers in this intervention allows them to focus on health domains case managers consider important, including adherence, depression, and substance use.

**Figure 1:**
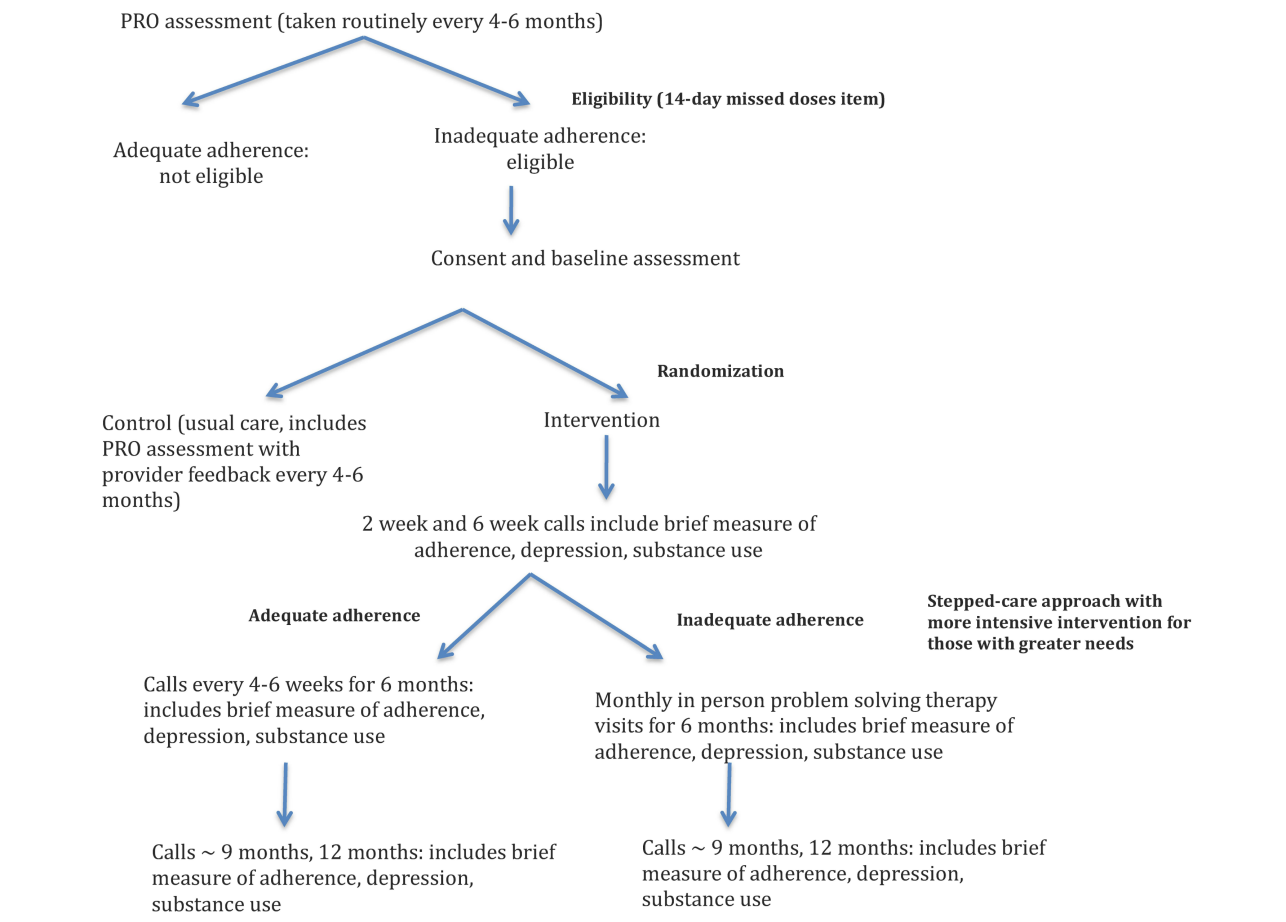
Participant flow including eligibility, enrollment, and follow-up. PRO: patient-reported outcomes.

Case managers are available for patient or case manager-initiated interactions in both the usual care and intervention arms and each case manager has patients in both arms. In addition, the intervention arm uses automated email reminders for case managers to facilitate a more systematic approach targeting those who need it most. All patients in the intervention arm receive a structured 10 to 15 minute follow-up telephone call from their case manager 2 and 6 to 8 weeks after enrollment. A brief assessment including the depression, medication adherence, and substance use instruments from the clinical assessment that is given to all patients as part of clinical care visits is administered at the beginning of each call. Case managers then provide education and support based on responses, working with patients to develop targeted goals and plans. After a second call, a stepped-care approach is used for those patients identified as needing more intensive interventions (Figure 1).

Patients in the intervention arm who continue to report inadequate adherence after 6 weeks receive a more intensive intervention based on the PEARLS approach, which includes (1) problem solving therapy, (2) social and physical activation, (3) pleasant events scheduling, and (4) patient support and education regarding ARV use. We modified this approach to facilitate clinical care integration and to accommodate daily experiences of PLWH. For example, we modified the list of possible pleasant events and activities taking into account the limited incomes of many patients. Motivational interviewing techniques are used to facilitate substance use reduction.

Problem solving therapy (PST) involves up to 6, approximately 40-minute sessions with the case manager over 6 months, typically in-person at the clinic. PST is a skills-enhancing behavioral treatment based on the assumption that the accumulation of problems in living cause and promote inadequate adherence (and other maladaptive behaviors and symptoms). PST is a skill-building method consistent with modern self-management support strategies used in managing chronic medical illness [78]. PST helps to define and clarify problems and provides a structured, realistic, and achievable approach to solve problems and meet individual goals. Everyone in the intervention arm including those doing well with adherence early on also receive follow-up “check-in” calls by the care manager with a brief assessment at approximately 9 and 12 months after enrollment.

#### Training

Case managers received a 2-day training session at the beginning of the trial focused on motivational interviewing techniques and PST led by an HIV specialist, psychiatrists, and psychologists with expertise in training for PST and motivational interviewing. An intervention manual based on IMPACT, PATHWAYS, and PEARL collaborative care trials formed the basis of training on collaborative care, stepped-care principles, and PST [50,55,57]. Training included didactics, role-playing exercises, group discussion of the role-play activities, and observation of a videotaped demonstration. We have also done 1 booster session to date and record a small subset of sessions looking for a means of improvement.

#### Platform

One goal was to use the PRO platform to promote practice system changes to improve outcomes by facilitating systematic monitoring and follow-up, proactive care management, and information sharing with the entire team. Based on this goal, we used the open-source PRO platform [79] already integrated into the clinic to collect the clinical assessment and used for similar work in oncology and other clinics [64,66,80-84]. The platform notifies team members by pager when a patient’s clinical assessment results suggest trial eligibility. Once enrolled and assigned to the intervention, the platform sends automatic email notifications to individual care managers when their patients are due for a call or PST session, tracks attempted and completed calls, and sends automatic reminders if calls or PST sessions are not completed. Care managers can use the platform to conduct brief phone-based assessments with patients at the beginning of follow-up calls. The platform incorporates skip patterns and eliminates paper-based forms. Finally, the platform facilitates rapid integration of care manager documentation including assessment results that can be directly entered into the UW HIV clinic’s electronic health records (EHR) to notify other health care team members as needed. The platform reduces research coordination time for the trial and promotes the feasibility of the intervention in clinical care and further provides an easy way to track intervention fidelity in terms of care manager completion rates.

#### Outcomes

The primary trial outcome is change in adherence as measured by the clinical assessment, however changes in depression symptom severity is also important. Standard PHQ-9 scores range from 0 to 27 and are categorized as: none (0-4 points), mild (5-9 points), moderate (10-14 points), moderately-severe (15-19), and severe (≥20 points) depressive symptom severity [70]. PHQ-9 standard scores have curvilinear measurement properties with respect to the latent trait of depression defined by all the items, meaning that a constant difference in score implies different amounts of depression symptom severity at different depression severity levels [85]. In this situation, using continuous standard total scores in regressions can lead to confusing and even biased findings [86]. We therefore will generate scores using item response theory (IRT) as done previously [85]. IRT-based depression severity scores have linear scaling properties with respect to the latent trait of depression defined by all the items [87]. These outcomes are based on clinical assessments integrated into routine care for all clinic patients and measured every 4 to 6 months, and do not include study specific measures. While using data from clinical care requires greater flexibility in terms of outcome timeframe windows, it allows data collection for primary outcomes to be completely distinct and independent of whether a patient was in the intervention or the usual care control arm.

Important secondary outcomes include antidepressant medication use, HIV-1 viral load (VL), alcohol and substance use, health-related quality of life, and symptom burden. Medications including antidepressant medication are collected as part of the EHR. Even medications filled by other pharmacies are captured because all prescriptions from the clinic are written within the EHR and then sent via the EHR to the correct pharmacy allowing a very high capture of medications including initiating a new antidepressant medication or increasing a dose of an existing antidepressant medication. VL values are measured as part of routine clinical care, and therefore timing varies. Cut-offs of <40 are considered undetectable although we also tend to repeat VL analyses using a cut-off of <400 to exclude viral blips. Health-related quality of life, symptom burden, and current drug and alcohol use are captured through the clinical assessment.

In addition, we will examine process outcomes, including provider diagnoses (depression, substance use) and referrals and visits with mental health counselors, substance use counselors, and health educators. Diagnoses including drug and alcohol use are captured in the EHR as is information regarding scheduled, kept, and cancelled visits as well as visit types. These outcomes are collected as part of routine care.

#### Incentives

There is no incentive for completing the clinical assessment as this is part of clinical care for all patients. Despite this, refusal rates have been low to date; the current refusal rate is 1%. There are no financial incentives for participating in this trial as the trial philosophy is assessing the impact of systematic approaches to care management to improve clinical care. Bus passes are available for participants who meet with their care manager on a day when they otherwise would not be in clinic.

#### Statistical Analyses

Clinical data will be used to assess differences between patients enrolled in the trial and eligible patients not enrolled. Primary analyses comparing outcomes in the usual care versus intervention arms will be performed on an intent-to-treat basis focused on 1 year after enrollment. Secondary analyses will be done on an as-treated basis with censoring if patients do not complete follow-up. We have previously found missing rates of <4% for clinical assessment items [64] but will use multiple imputation approaches to address this if necessary. Descriptive analyses compare patients in the intervention and usual care arms on demographic and clinical variables, including depression status, adherence, substance abuse, use of mental health services, and use of antidepressant medications to assess whether groups are balanced at baseline. Depending on the distribution, we will likely use linear mixed-effects models [88,89] to assess differences in adherence levels taking into account within-subject correlations between adherence levels over time and the influence of potential confounding covariates. We will look for influential outliers in the regression model and we will explore nonlinear transformations of the outcome measure to see if the linearity assumption is violated. We will check an unstructured version of the working correlation matrix to ensure that our default working correlation, exchangeable, is properly specified. Selection of variables for inclusion in the models will be based on likely confounding factors based on the literature to date, coupled with inclusion of variables observed to be strong risk factors for the outcome and are therefore potential “precision variables” to improve statistical efficiency. Model fit will be assessed with *R*^2^, we will report the ratio between the variance that is accounted for by the random effects and the residual variance, and we will determine whether the linear mixed-model’s distributional assumptions are met [90]. We will examine effect modification of the pattern of change in adherence over time between groups by depression and substance abuse status. We will also examine baseline characteristics as potential moderators of intervention effects [91,92]. We will compare loss-to-follow-up rates in the 2 groups. If these rates exceed 5% or differ between groups, we will use baseline assessment data and clinical information data to assess for potential bias. Sensitivity analyses will be conducted to determine the impact of dropout [93,94] and spillover effects and to evaluate additional longer timeframes. We will examine the impact of the intervention on visit frequency with case managers in both the intervention and usual care arms looking for potential spillover effects of the intervention on the usual care arm. Our last sensitivity analysis will be an “as treated” analysis, using inverse probability of treatment weights. This will account for the possibility of differential switching between groups (ie, drop out) as a form of contamination and can assist in the interpretation of the primary endpoint. However, we intend to use the intention to treat analysis as the primary study endpoint, as we see the benefits of randomization for confounder control as exceeding the risks of validity posed by contamination. We thus follow recommendations of a Cochrane review: randomized design, follow patients for >5 months, analyze patients in the groups to which they were randomized, and control for baseline values or use differences between follow-up and baseline values as the main outcome [95].

#### Sample Size

We based sample size estimates on a two-tailed significance level (alpha) of .05 and power (1-beta) of .80 [96]. With 115 individuals in each arm (N=230), we would be able to detect even a 4% increase in adherence in the intervention arm. We expect a loss to follow-up rate between 8% and 10%, but conservatively account for a 15% loss to follow-up rate and increased our overall sample size target to 270. Power for this type of trial should be sufficient to identify small to moderate effects [97] (defined as effect sizes of 0.2-0.5 [98]). Even if we have a higher than expected loss to follow-up rate of 15%, we will have sufficient power to detect effect sizes of 0.35, well within the small-to-moderate effects continuum and in line with the magnitude of effects seen in other adherence intervention studies [44,99,100]. We expect several of our outcomes will be more responsive to change than overall adherence particularly many of the binary process outcomes. We will have more than sufficient power to detect differences in those outcomes. While we plan to attempt to enroll 270 individuals, one of the outcomes of interest is the willingness of patients to participate in these types of interventions. Therefore, we are also specifically interested in whether it is feasible to enroll this many individuals from a clinical care setting in a reasonable amount of time.

#### Trial Registration, Ethics, Consent, and Institutional Review Board Approval

This trial is registered in clinicaltrials.gov (NCT01505660) and received approval from UW Human Subjects Division (UW #41128). Informed consent to participate in the study was obtained from each participant. They are not pressured to participate and participation or not does not impact their ability to receive care at the clinic.

## Results

The National Institutes of Health funded this study and had no role in study design, data collection, or decisions regarding whether or not to submit manuscripts for publication. This trial is currently underway with follow-up time still accruing.

## Discussion

### Design

The key consideration in designing this trial was to adapt and test an evidence-based model of collaborative care [50] to enhance ARV adherence. We used existing clinical staff members in a manner that is streamlined and could be integrated into care. The intervention offers patients and health care delivery teams resources necessary to increase the use of evidence-based treatments to improve adherence, depression, and substance use. The case manager-based collaborative model is a system of care that can be integrated into clinical care settings to provide patient-centered care. The intervention was modeled on the PEARLS and PATHWAYS studies [50,51] based on the IMPACT study [57]. We made modifications to focus on adherence and care for PLWH and to ensure that the interventions were sufficiently streamlined to facilitate use in routine care.

### Stepped-Care Approach

We are using a stepped-care approach allowing intervention intensity to be tailored to the patient’s needs [101]. Earlier depression studies suggested incorporating additional interventions led to improvements and sustainability beyond that obtained by telephone care management alone [102-104]. Similarly, adherence studies have demonstrated a greater impact with added services targeting those with the highest needs [45]. This stepped-care approach allows us to target these interventions to those at greatest need and who are most likely to benefit rather than on everyone to maintain feasibility for incorporating into busy clinical care settings.

### Telephone

While many care management protocols incorporate community outreach and home visits, these are not likely applicable to HIV care in the current funding environment. However, telephone-based care management approaches [53,54,104-107] have shown improvements in readmission rates and depression levels. Therefore, we included a brief telephone-based approach for the initial interactions (2 and 6 weeks), as well as later check-ins for those doing well, to enhance feasibility.

### Intensity

We designed the intervention to be less intensive than previous care management trials [50,51,63] to enhance feasibility in clinical practice. Prior care management approaches often included home visits and frequent, hour-long sessions for everyone in the intervention group. We truncated these practices and are using phone-based check-in calls, and office- or phone-based visits for PST targeted to those with the greatest need. An advantage of using case managers to deliver the intervention is the ability to leverage existing patient-case manager relationships.

### Adherence Measurement

Patients with inadequate medication adherence measured by the 14-day adherence item are eligible for inclusion. Although self-reported adherence has been critiqued for inaccuracies, these concerns have centered on over-reporting rather than under-reporting of adherence. Patients who report poor adherence are likely to truly have poor adherence. We chose the 14-day timeframe to include weekends, which are frequent times of missed doses and avoided longer time frames to limit recall errors and focus on current behavior.

### Limitations

This intervention is being evaluated in English-speaking patients only. A Spanish language version of the clinical assessment is now offered at the UW HIV clinic, and an Amharic version has just been introduced. Another limitation is it includes only one clinic. Additional studies will be needed at other sites and in non-English speaking patients to determine if findings are generalizable. While we track the number of call attempts (successful and unsuccessful) and other contributions to staff time burden, our focus is not specifically on evaluating costs. The use of self-reported adherence as one of the primary outcomes may be considered a limitation. We have previously demonstrated high correlations between self-reported adherence and viral load, pharmacy refill data, and unannounced pill count data [108,109]. Furthermore, self-reported adherence data has been shown not to inflate effects of adherence interventions [44]. Self-reported adherence is one of several outcomes. We collect adherence in the clinical assessment from all patients as part of care as opposed to just in the intervention itself, reducing the likelihood of biased responses from those in the intervention group. The clinical assessment includes a normalizing statement, and we selected an intermediate timeframe to minimize concerns with recall bias. A prior meta-analysis has demonstrated that the ability to identify existing intervention effects are stronger when using adherence measures with longer recall periods (2 weeks or 1 month rather than 7 days or shorter) [44].

Another potential limitation is that providers and case managers can have patients in both usual care and intervention arms, and therefore may be subject to spillover effects, which could dilute the impact detected. Systematic changes such as automated email reminders of time to contact patients are not subject to spillover. Concern about spillover effects is one of the factors that led some investigators to evaluate nonintegrated approaches for delivering interventions. However, given the potential advantages of having a care manager with an established relationship with the patient, as well as designing an intervention that would be feasible within clinical care settings, we elected to conduct this intervention using existing clinic case management staff despite the potential for spillover effects. While this does not allow feasible blinding, the outcomes are assessed as part of routine care and not part of the trial.

Finally, the intervention is a comprehensive multifaceted approach, so we may be unable to isolate the critical factors necessary for success.

### Strengths

A key strength is the adaption of an evidenced-based collaborative care intervention for use among PLWH with inadequate adherence. A collaborative care approach has long been recommended for improving adherence [110], as has a stepped-care approach targeting patients with particular difficulty to receive more intensive strategies [110]. We are attempting to maximize generalizability by conducting this study among a clinical care patient population. We identify patients from the clinical assessment given to the entire clinic population as part of routine care. This approach contrasts with the subset of patients who return mail-in surveys, which has often been the recruitment strategy for other care management studies. In particular, we do not exclude patients with substance abuse or depression. In fact, these two inter-related problems are key contributors to inadequate adherence in clinical care, and therefore this intervention addresses all three rather than focusing on inadequate adherence in isolation. Another strength is using care managers who are routinely part of HIV care settings as this makes it much more likely to be feasible for widespread use.

We integrated proactive telephone check-ins. There is considerable support for the efficacy of telephone-based interventions with a number of health problems in various patient populations [111]. Among PLWH, recent studies have focused on sexual risk reduction [112], interpersonal therapy for rural PLWH with depression [113], tobacco cessation [114], and adherence [115]. Potential advantages of telephone-based strategies include cost effectiveness and reducing barriers to care [116]. We optimized resource allocation by combining routine telephone check-ins with in-clinic, in-person PST for those who need a more intensive intervention.

Finally, using a Web-based PRO platform for data collection enhances fidelity, and allows automation of reminders to case managers and a more proactive systematized approach to routine follow-up.

### Conclusion

Medication adherence is critically important for long-term outcomes among PLWH. There is a need to identify best practices for implementing evidence-based collaborative care models that are effective and feasible in clinical care. Adherence efficacy trials have not led to sufficient improvements, and there remains little guidance regarding how adherence interventions should be implemented into clinical care. By focusing on improving adherence within care settings using existing staff, routine assessment of key domains, such as depression, adherence, and substance use, and feasible interventions, we propose to evaluate this innovative way to improve clinical outcomes.

## Abbreviations

ARVs: antiretroviral medications
EHR: electronic health records
HIV: human immunodeficiency virus
IRT: item response theory
PEARLS: Program to Encourage Active, Rewarding Lives study
PHQ-9: Patient’s Health Questionnaire
PLWH: persons living with HIV
PROs: patient-reported outcomes
PST: problem solving therapy
RCT: randomized controlled trial
UW: University of Washington
UW HIV: University of Washington Harborview Madison HIV
VL: viral load
